# An experimental study on the impact of clinical interruptions on simulated trainee performances of central venous catheterization

**DOI:** 10.1186/s41077-017-0038-1

**Published:** 2017-02-14

**Authors:** Jessica Jones, Matthew Wilkins, Jeff Caird, Alyshah Kaba, Adam Cheng, Irene W. Y. Ma

**Affiliations:** 1 W21C, University of Calgary, Calgary, Canada; 2 Department of Psychology, University of Calgary, Calgary, Canada; 3 Faculty of Law, University of British Columbia, Vancouver, Canada; 4 Department of Community Health Sciences, University of Calgary, Calgary, Canada; 5 Department of Pediatrics, University of Calgary, Calgary, Canada; 6 Department of Medicine, University of Calgary, 3330 Hospital Dr NW, Calgary, AB T2N 4N1 Canada

**Keywords:** Central venous catheterization, Attention, Interruption, Task performance and analysis, Medical errors

## Abstract

**Background:**

Interruptions are common in the healthcare setting. This experimental study compares the effects of interruptions on simulated performances of central venous catheterization during a highly versus minimally complex portion of the task.

**Methods:**

Twenty-six residents were assigned to interruptions during tasks that are (1) highly complex: establishing ultrasound-guided venous access (experimental group, *n* = 15) or (2) minimally complex: skin cleansing (control group, *n* = 11). Primary outcomes were (a) performance scores at three time points measured with a validated checklist, (b) time spent on the respective tasks, and (c) number of attempts to establish venous access.

**Results:**

Repeated measure analyses of variances of performance scores over time indicated no main effect of time or group. The interaction between time and group was significant: *F* (2, 44) = 4.28, *p* = 0.02, and partial eta^2^ = 0.16, indicating a large effect size. The experimental group scores decreased steadily over time, while the control group scores increased with time. The experimental group required longer to access the vein (148 s; interquartile range (IQR) 60 to 361 vs. 44 s; IQR 27 to 133 s; *p* = 0.034). Median number of attempts to establish venous access was higher in the experimental group (2, IQR 1–7 vs. 1, IQR 1–2; *p* = 0.03).

**Conclusions:**

Interruptions during a highly complex task resulted in a consistent decrement in performance scores, longer time required to perform the task, and a higher number of venous access attempts than interruptions during a minimally complex tasks. We recommend avoiding interrupting trainees performing bedside procedures.

## Background

Interruptions in healthcare are common and occur ubiquitously. In an observational study of an intensive care unit, interruptions of healthcare professionals occurred at a rate of 14 times per hour [1], while physicians and nurses in the emergency room setting in a trauma center were observed to be interrupted more than ten times per hour [2]. In the operating room, a mean of 50 interruptions was noted per case, [3] and on the medical ward, interruptions were also frequently present [4–6].

Given the limited working memory of individuals [7, 8], the impact of interruptions is such that, once interrupted, individuals may forget to resume the original task [9], take longer to complete the task [10], or complete tasks with higher error rates [11–13]. Overall, the yearly cost of interruptions to the hospital has been estimated to be more than US$51,000 per hospital [14]. With over 5600 hospitals in the USA [15], interruptions are estimated to contribute to costs of over US$280 million per year.

Central venous catheter (CVC) insertion is a commonly performed procedure. In the USA, an estimated 20.1 million central-line days per year occur on inpatient wards [16]. Although indicated for many medically ill patients, CVCs have associated complication risks, with an estimated complication rate of over 15% [17]. The insertion of CVCs is a challenge for many trainees, as the procedure involves multiple steps [18, 19]. Further, while the use of ultrasound guidance is intended to improve patient safety [20, 21], its use adds to the complexity of the procedure.

Complex tasks have previously been shown to be more susceptible to the effects of interruptions than simpler tasks [22], and less experienced trainees are more susceptible to interruptions than experienced physicians [23]. Given that many trainees at teaching centers perform ultrasound-guided CVC insertions [24], we hypothesize that trainees may be quite susceptible to interruptions and that disruption of their attentional focus may compromise procedural performances, especially during critical aspects of the task. As such, this study, via the use of simulation, seeks to examine the impact of interruptions at two different time points in the procedure performed by medical trainees and evaluates the impact of these interruptions on procedural performances. Specifically, we hypothesize that interruptions during a task that is complex, compared to interruptions during a task that is low in complexity, will result a lower procedural performance score, longer time spent on the procedural task, and a higher number of attempts to establish venous access.

## Methods

### Participants

All internal medicine residents (*n* = 98; postgraduate year (PGY)-1 to PGY-5) were invited to participate in this study between December 2012 and October 2013.

### Protocol

**Fig. 1:**
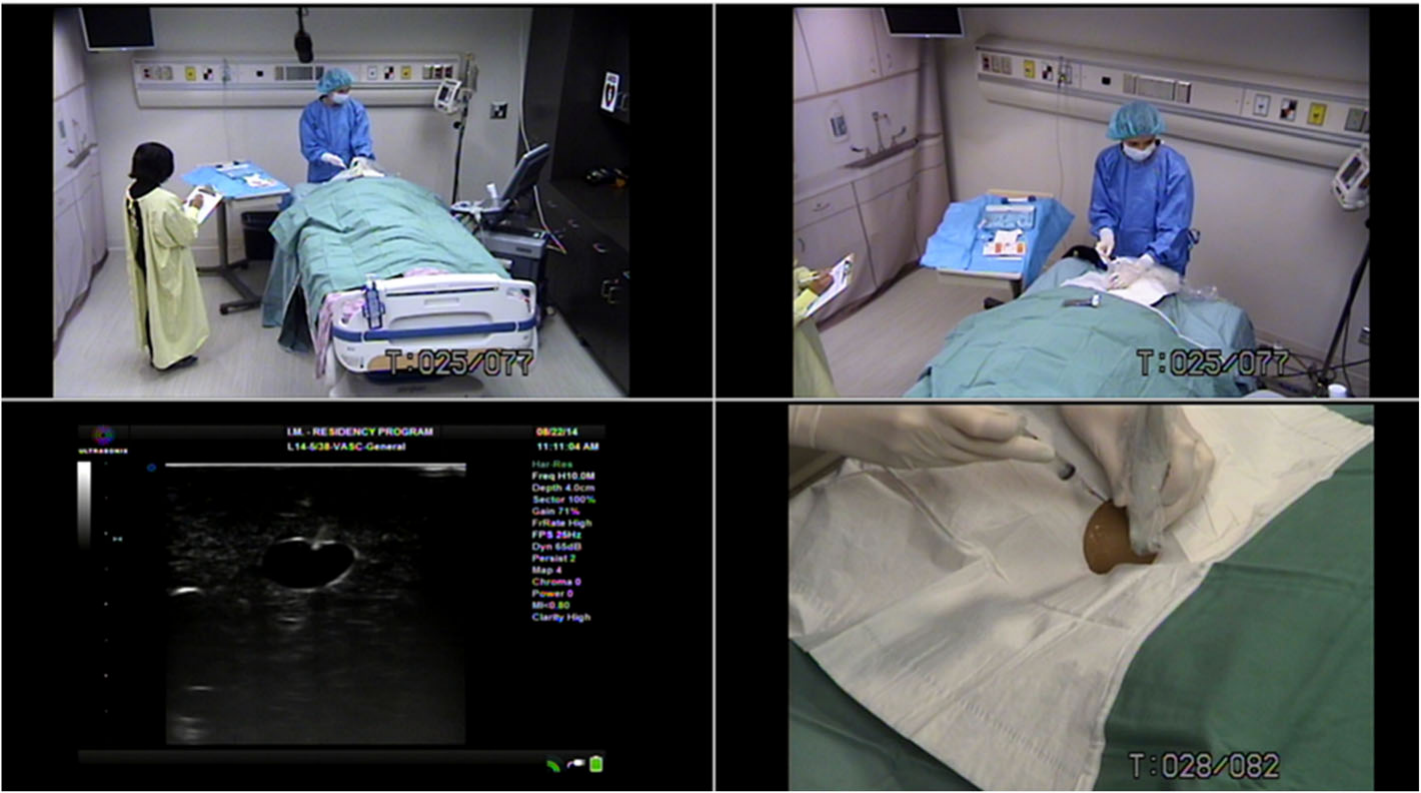
The four camera views of the central venous catheterization procedure, from the foot of the bed (*upper left*), from the right showing the procedural tray (*upper right*), from the left showing the procedural site (*lower right*), and the ultrasound screen (*lower left*)

At baseline, all consenting participants completed a demographic survey. Participants were then given standardized instructions to place a CVC into the right internal jugular (IJ) vein using ultrasound guidance (SonixTOUCH, BK Ultrasound©) on a simulator (Gen II Ultrasound Central Line Training Model, Blue Phantom™), in a standardized procedure room. The participants were informed that the patient had chronic kidney disease and no peripheral intravenous access. One confederate nursing assistant was in the procedure room and provided assistance as requested by the participants. During the procedure, participants communicated with the patient, whose voice was controlled by researchers in the adjacent control room behind a one-way mirror. The scenario was video recorded using four camera angles, capturing views of the room, procedure site, procedural tray, and ultrasound screen (Fig. 1). Postprocedure, all participants underwent a 30-min semi-structured interview on their strategies for dealing with interruptions.

### Interruption

Participants were assigned to two groups in this study. Due to accidental violations in the randomization procedure, the majority of the participants (85%) were not randomized but assigned using unconcealed alternating group assignment. In the control group, participants were interrupted during a task that was felt to be low in complexity: skin cleaning for the insertion site. In the experimental group, participants were interrupted during a more complex task: establishing venous access under direct ultrasound guidance, where the interruption occurred as soon as the venous access needle entered the simulated skin.

At the pre-defined task (i.e., at the time of skin cleaning for the control group and at the time of venous needle skin entry for the experimental group), a 5-s interruption was introduced by a telephone call into the procedure room, whereby the nursing assistant relayed the message on the patient’s high potassium (7.9 mmol/L). An electrocardiogram indicating clinical severity (e.g., peaked T waves and widened QRS) was available if requested by the participant. The nursing assistant was instructed to acknowledge all orders from the participants except for intravenous orders, whereby the participants were reminded that the patient had no intravenous access. As therapy for the hyperkalemia requires intravenous access, it is anticipated that the participants would need to complete the CVC task.

### Outcomes

The primary outcomes of interest were (1) overall performance of CVC insertion, (2) time spent on the respective tasks, and (3) number of attempts to establish venous access. Secondary outcomes included results from the thematic analyses of the interviews.

#### Performance of CVC insertion

Performance of CVC insertion was assessed using a 23-item checklist, modified from a previously published tool with validity evidence, to ensure that the items were applicable to our current task [25, 26].

Items that were executed appropriately were given a score of two, items that were not completed were given a zero, while items that were completed inappropriately or suboptimally were given a score of one. From this checklist, four scores were generated, presented as a percentage:

1. Overall score: sum of checklist score.
2. Time 1 score: steps prior to and including cleaning.
3. Time 2 score: steps after cleaning until venous access establishment.
4. Time 3 score: remaining steps in the procedure.

All performances were rated by a faculty (IM) with over 10 years of prior experience in rating CVC performances and previously demonstrated high inter-rater reliability using a similar tool [26]. Blinding of the rater to group assignment was not possible as the videos clearly indicate when the interruptions occurred.

#### Time spent on procedure

*Cleaning time* was defined as the time taken to clean the insertion site. *Time required to access the IJ vein* was defined as the time from first needle puncture until successful venous access, as indicated by the removal of the syringe for wire insertion.

#### Number of attempts

The number of attempts taken to establish venous access using the needle and syringe was recorded. Number of attempts was recorded independently by two researchers (IM and MW). Inter-rater reliability for this measure was high [intraclass correlation coefficient = 0.97, 95% confidence interval (CI) 0.93 to 0.99].

### Statistical analyses

Group differences were compared and analyzed in an intention-to-treat basis using standard parametric and non-parametric techniques [27]. Construct validity of the checklist was assessed by comparing performance scores of junior trainees (PGY 1–2) with senior trainees (PGY 3–5): 71.7 ± standard deviation (SD) 22.8 vs. 88.1 ± 5.9%, respectively; *p* = 0.028. Internal reliability of the checklist was assessed using Cronbach’s alpha (alpha = 0.88).

After testing for the assumption of sphericity (not violated, chi-square (2) = 0.89, *p* = 0.64, epsilon = 0.96), mixed repeated measure analyses of variances were conducted to assess for group differences on performance scores on the three time points. Partial eta squared values are reported as measures of effect sizes and interpreted as follows: <0.01 = small effect, <0.06 = medium effect, and >0.14 = large effect [28]. Significant interaction between group and time was further explored using the Bonferroni adjustments.

All performances were recorded and time coded with Noldus Recorder and Observer XT, version 11.0 (Noldus Information Technology, Wageningen, the Netherlands). All analyses were performed using SAS 9.3 (SAS Institute Inc., Cary, NC) and PASW Statistics, version 18.0 (PASW, IBM Corporation, Somers NY).

### Qualitative data analyses

Interview data was transcribed into NVivo, version 10 (QSR International, Burlington, MA). Thematic content analysis was performed independently by two researchers (IM, JJ) [29]. Assigned codes were reviewed and coded several times to ensure the saturation of themes. Codes were then grouped together based on similarities and linkages to form broader categories. Agreement in coding was high (Kappa = 0.89; 95% CI 0.87 to 0.90) [30]. Disagreement in coding was resolved by consensus.

## Results

Twenty-six participants completed the study protocol. Of these, 11 (42%) to the control group and 15 (58%) were assigned to the experimental group. There were no significant demographic differences between the two groups (Table 1).

**Table 1:** Baseline characteristics of 26 participants^a^

Baseline characteristic	Control (skin cleansing) group *n* = 11	Experimental (venous access) group *n* = 15	*p* value
Postgraduate year level^b^			
1 and 2	5 (56)	8 (62)	1.00
3 to 5	4 (44)	5 (38)	–
Gender			
Males	9 (82)	9 (60)	0.39
Females	2 (18)	6 (40)	–
Months rotating in the intensive care unit			
0 or 1	7 (64)	7 (47)	0.39
2 or more months	4 (36)	8 (53)	–
Median no. of central venous catheterization performed (interquartile range)	3 (1–12)	3 (1–25)	0.51
Mean self-rated ability to perform procedure (± standard deviation)^c^	2.5 ± 1.2	2.9 ± 1.4	0.48

^a^Data presented as number (percentage) unless otherwise indicated

^b^Missing values occurred because not all participants answered all questions

^c^Rated out of 6; where 1 = not competent to perform independently, 6 = above average to perform independently

### Performance outcomes

**Fig. 2:**
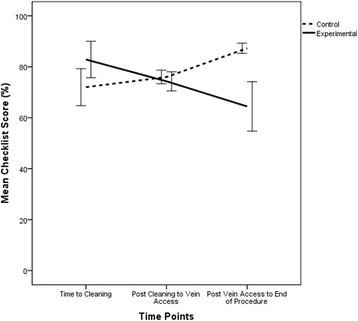
Mean checklist scores at three time points for participants in the control (skin cleansing) group (*n* = 11) and participants in the experimental (venous access) group (*n* = 15). *Error bars* indicate standard error

Overall checklist scores did not differ between groups (control group 82.7 ± SD 8.7% vs. experimental group 72.6 ± 23.4%; *p* = 0.16). Scores for the three time points are shown in Fig. 2. There was no significant main effect of time or group assignment: *F* (2, 44) = 0.08, *p* = 0.92, partial eta^2^ = 0.004; *F* (1, 22) = 0.46, *p* = 0.50; partial eta^2^ = 0.021, respectively. However, the interaction between time and group assignment was significant: *F* (2, 44) = 4.28, *p* = 0.02, partial eta^2^ = 0.16, indicating a large effect size.

Post-hoc pairwise comparisons were not significant (mean score difference [experimental group score – control group score] at time 1: 10.9 ± SD 10.5%, *p* = 0.31; time 2: −1.71 ± 5.0%, *p* = 0.73; time 3: −22.8 ± 11.7%, *p* = 0.06).

### Time spent on procedure

Mean *cleaning time* did not differ between the two groups (control group 43 ± 17 s vs. experimental group 37 ± 13 s; *p* = 0.33).

Median *time required to access the IJ vein* was significantly longer in the experimental group (148 s; interquartile range [IQR] 60 to 361 vs. 44 s; IQR 27 to 133 s; *p* = 0.034).

### Number of attempts

The median number of attempts to establish venous access was significantly higher in the experimental group (2, IQR 1–7) than that of the control group (1, IQR 1–2), *p* = 0.03.

### Impact of interruptions

Two participants in the experimental group (13%) punctured the carotid artery while none in the control group did so. In the experimental group, technical errors observed included: one participant failed to aspirate during needle advancement while dealing with the interruption. Before and after the interruption, this participant did not display this suboptimal needle advancement technique. One participant left the needle open to air while managing the interruption. These errors were not observed in the control group.

In the control group, two participants cleaned the same area twice with the same sponge during the interruption. Another participant missed cleaning the center of the target area while being interrupted. These suboptimal cleaning techniques were not displayed during the first cleaning attempt before the interruption, nor were these techniques observed in the experimental group.

### Thematic analysis

In the interview, participants reported being interrupted, outside of this study, a median of once per procedure (IQR 0 to 2). Analyses from semi-structured interviews revealed that the participants reported using an average of 4 ± SD 2 strategies in managing interruptions (Table 2). Task prioritization was the most commonly reported strategy (*n* = 19; 73%).

**Table 2:** Strategies used to manage interruptions, as reported by the 26 participants

Strategy	No. (%) reported using strategy	Example
Internal strategies		
Talk aloud	1 (4)	“Talking out loud: where am I, what is next?”
Reorienting (not specified)	7 (27)	
Mental Checklist	4 (15)	“Think about the order of the procedure in my mind. Continue to go through it and go back to the list.”
Recap last steps	4 (15)	“Retrace the last few steps, the last three things, this is where I need to go.”
Mental bookmarking	2 (8)	“Bank your thoughts – try not to lose my spot in what I was doing.”
Physical layout of equipment	1 (4)	“Lay things out so I know where I am.”
Focus (not specified)	6 (23)	
Prioritizing tasks	19 (73)	“The most pressing issue is the one I will address.”
Concentrating on one thing at a time	9 (35)	“Focus on one thing at a time. Not good at multi-tasking.”
Delegating tasks	8 (31)	“Delegate to the clerk.”
Stop and think	6 (23)	“Stop and decide if you should continue. Stop everything in a safe position and decide.”
Ignoring interruption	2 (8)	“Tried to ignore it initially – questioned if I should continue with the procedure.”
Stay calm	2 (8)	“Try to remain calm.”
Maintaining accuracy of primary task	1 (4)	“Doing what needed to be done – do it right.”
Mental chunks	1 (4)	“Manage in moments, split up your work into manageable chunks.”
Multitask	2 (8)	“It was distracting – I didn’t have full attention on either task. Trying to do both…had 80% attention on the procedure.”
External Strategies		
Hurry primary task	3 (12)	“Made me do it faster.”
Handing over pager	3 (12)	“Carry the pager for [those doing procedures]”
Communication (not specified)	2 (8)	

## Discussion

Our study identified that although performance scores do not differ between groups, interruptions during the experimental condition resulted in a number of serious procedural errors that were not observed in the control group. Examples of these errors included carotid puncture, leaving the needle open to air, and failure to aspirate during needle insertion. Further, a number of errors in cleaning technique were observed in the control group. These errors were likewise not observed in the experimental group, nor were they observed in the control group prior to the interruption.

Prior studies have examined the impact of interruptions on tasks such as peg or object transfer tasks and artificial distraction tasks, such as performing arithmetic [10, 31–33]. In these studies, distractions typically were shown to result in a decrease in the performance of the distracting task [31], the primary task [10, 32], or both [33]. However, there remains a need for further research on the impact of interruptions on clinical outcomes [34–37]. To our knowledge, our study is the first to explore the impact of interruptions using primary and interruption tasks specific to the CVC procedure, a complex procedure that is commonly performed [16]. Our results identified technical errors as a result of interruptions. Further, the decrement in performance, time taken, and number of attempts made was significantly worse when the interruption occurred at a more complicated part of the procedure. Although our participants reported employing multiple strategies to manage the impact of interruptions, our results suggest that these strategies may be ineffective at preventing the negative consequences of interruptions. As such, limiting interruptions for trainee performances of CVC may be warranted [37].

Our study has a number of limitations. First, as a single-centered study, the generalizability of our conclusions may be limited. Our trainees were relatively inexperienced overall. Therefore, our results do not pertain to experienced proceduralists. Second, we did not assess the performance on the interrupting task itself. Potentially, participants whose CVC tasks suffered the most may have dealt with the interrupting task the best. However, since effective treatment for hyperkalemia required an intravenous access, and none of the participants chose to place an over-the-needle catheter into the IJ (all chose to complete the entire CVC insertion), performance on the interrupting task would have been immaterial. Third, although our group assignment was initially intended to be randomized, due to accidental violations in the randomization procedure, the majority of the participants were ultimately assigned in a non-randomized fashion (i.e., alternating group assignment). However, baseline participant characteristics were similar in both groups and no significant baseline differences were found in the two groups. Nonetheless, our study was not randomized in nature and therefore, we cannot exclude the possibility that the two groups systematically differed from each other. Fourth, our raters were not blinded to the group assignment and therefore are subject to potential bias. Further, we had duplicate raters only for some, not all, outcome measures. However, on those measures, our inter-rater reliability was high. Fifth, we did not perform a sample size calculation. We ultimately were only able to recruit a convenience sample of 26 participants, due to scheduling and availability issues and the voluntary nature of our study. Future studies should consider an a priori sample size calculation based on a single primary outcome. Sixth, we were unable to determine the exact time delay attributable to the cognitive effects of the interruptions, as we did not ask the participants to perform a think-aloud protocol. Future studies may consider the use of such a protocol. Seventh, we were unable to detect a difference in performance scores between groups, which may be a result of using a checklist which tended to award points for completing steps successfully [38]. Potentially, the use of a checklist that specifically assesses for errors may be better suited to detect performance issues that arose as a result of the interruptions [39].

These limitations notwithstanding, overall, our study demonstrated that CVC performances are significantly impaired by interruptions, especially during a highly complex task. We therefore argue for the need to prevent these interruptions and/or develop effective strategies to assist trainees in mitigating the negative effects of interruptions on procedural performances. Previous studies have shown that visible signage and checklists may reduce the incidence of interruptions [37, 40]. In another study, the implementation of a “no interruption zone” for nurses resulted in a significant decrease in the number of interruptions [41]. For CVC insertions, pager hand-off could be considered to help minimize interruptions [42]. Nonetheless, some degree of interruption in healthcare may be unavoidable. As such, systems-wide strategies for reducing the impact of interruptions need to be examined, and educators should consider training learners to deal with procedural interruptions.

## Conclusions

CVC performances are significantly impaired by interruptions. We recommend that trainees performing CVC insertions should not be interrupted during the procedure, especially during highly complex tasks.

## Abbreviations

CI: Confidence interval
CVC: Central venous catheter
IJ: Internal jugular
IQR: Interquartile range
PGY: Postgraduate year
SD: Standard deviation
